# Osteogenic Response to Polysaccharide Nanogel Sheets of Human Fibroblasts After Conversion Into Functional Osteoblasts by Direct Phenotypic Cell Reprogramming

**DOI:** 10.3389/fbioe.2021.713932

**Published:** 2021-09-03

**Authors:** Kei Nakai, Kenta Yamamoto, Tsunao Kishida, Shin-ichiro Kotani, Yoshiki Sato, Satoshi Horiguchi, Hironaka Yamanobe, Tetsuya Adachi, Francesco Boschetto, Elia Marin, Wenliang Zhu, Kazunari Akiyoshi, Toshiro Yamamoto, Narisato Kanamura, Giuseppe Pezzotti, Osam Mazda

**Affiliations:** ^1^ Department of Immunology, Graduate School of Medical Science, Kyoto Prefectural University of Medicine, Kyoto, Japan; ^2^ Department of Dental Medicine, Graduate School of Medical Science, Kyoto Prefectural University of Medicine, Kyoto, Japan; ^3^ Ceramic Physics Laboratory, Kyoto Institute of Technology, Kyoto, Japan; ^4^ Department of Polymer Chemistry, Graduate School of Engineering, Kyoto University, Kyoto, Japan

**Keywords:** nanogel, polysaccharide, fibroblast, cell reprogramming, osteogenic, osteoblast, direct conversion

## Abstract

Human dermal fibroblasts (HDFs) were converted into osteoblasts using a ALK inhibitor II (inhibitor of transforming growth factor-β signal) on freeze-dried nanogel-cross-linked porous (FD-NanoClip) polysaccharide sheets or fibers. Then, the ability of these directly converted osteoblasts (dOBs) to produce calcified substrates and the expression of osteoblast genes were analyzed in comparison with osteoblasts converted by exactly the same procedure but seeded onto a conventional atelocollagen scaffold. dOBs exposed to FD-NanoClip in both sheet and fiber morphologies produced a significantly higher concentration of calcium deposits as compared to a control cell sample (i.e., unconverted fibroblasts), while there was no statistically significant difference in calcification level between dOBs exposed to atelocollagen sheets and the control group. The observed differences in osteogenic behaviors were interpreted according to Raman spectroscopic analyses comparing different polysaccharide scaffolds and Fourier transform infrared spectroscopy analyses of dOB cultures. This study substantiates a possible new path to repair large bone defects through a simplified transplantation procedure using FD-NanoClip sheets with better osteogenic outputs as compared to the existing atelocollagen scaffolding material.

## Introduction

Modern challenges toward a super active aging society require diversification of the available options for bone regeneration therapies, which include large-scale bone defects caused by bone diseases (Gibon et al., 2016). Large-scale bone defects are generally associated with various bone diseases caused by resection of bone tumors, failure in repairing osteoporotic fractures, rheumatoid arthritis, alveolar bone resorption induced by severe periodontal disease, osteomyelitis, and intractable diseases caused by medication-related osteonecrosis of the jaw in patients who received bone-modifying agents (Limones et al., 2020). Osteoblasts produce bone matrix, contribute to bone formation, and play a central role in bone remodeling; their proliferation and activity are essential for the recovery of bone defects (Kim et al., 2020). However, osteoblasts generally possess low proliferative capacity and such an inherently low capacity might significantly decrease with aging (Boskey and Coleman, 2010). In such eventuality, external supply by cell transplantation might help to promote bone regeneration.

In recent years, a number of reports have been published on bone regeneration treatments using mesenchymal stem cells (Wang et al., 2013; Liu et al., 2014; Jin and Lee, 2018). Other reports suggest the possibility of using induced pluripotent stem (iPS) cells or dental pulp stem cells to induce osteoblasts (Graziano et al., 2008; Rana et al., 2019). An alternative technique is direct reprogramming, or direct conversion, that induces a phenotypic change of somatic cells (Ieda et al., 2010; Kim et al., 2011; Nizzardo et al., 2013). The successful direct conversion was reported from human fibroblasts to functional osteoblasts, brown adipocytes, Schwann cells, urothelial cells, and so on (Kishida et al., 2015; Yamamoto et al., 2015; Yamamoto et al., 2016; Sowa et al., 2017; Wakao et al., 2017; Sato et al., 2018; Yamamoto et al., 2018). In both bone transplantation and bone regeneration treatment, the direct conversion method could potentially be useful when applied with autologous osteoblasts. However, in order to heal large-scale bone defects or bone resorption sites, it is necessary to retain the transplanted cells within the bone defect, which in turn requires the preparation of an appropriate scaffold to be transplanted together with the induced osteoblasts. Producing and maintaining a dense population of osteoblasts necessarily requires the use of high-quality scaffolds. This study attempted to satisfy the requirements of a dense cell population of human fibroblasts to be directly converted into osteoblasts on engineered freeze-dried nanogel-cross-linked porous (FD-NanoClip) polysaccharide scaffolds with different morphologies.

Nanogel consists of nanometer-sized polymer nanoparticles with 3D networks formed by the cross-linking of polymer chains (Hashimoto et al., 2015; Hashimoto et al., 2018). Nanogel-cross-linked porous (NanoClip) gel has been used as a scaffold for tissue engineering, including bone regeneration, which is biocompatible and biodegradable and has an interconnected porous structure that can supply oxygen and nutrients to cells. Additionally, the NanoClip gel trapped proteins and liposomes *via* hydrophobic interactions because its amphiphilic nanogels exhibit chaperone-like activity. Cellular microenvironments can be achieved by using scaffolds, growth factors, or a combination of the two. Nanogels serve as an artificial extracellular matrix (ECM) for facilitating cell adhesion and cell proliferation and as a reservoir for maintaining growth factors. This suggests that NanoClip gel is a promising scaffold for tissue engineering and a versatile scaffold for efficient bone formation (Hashimoto et al., 2015; Hashimoto et al., 2018).

In a previous report (Sato et al., 2018), FD-NanoClip polysaccharide was used as a scaffold material and osteoblast-like cells in three-dimensional culture generated by means of a direct conversion technology that transfers genes into human fibroblasts using a retrovirus vector. According to this approach, HDFs were phenotypically converted into osteoblasts, in the scaffold where the bone matrix was significantly formed. However, this approach involves a risk of tumorigenesis because the preparation of osteoblast-like cell complexes requires the use of a retrovirus vector. In a successive study (Yamamoto et al., 2018), a successful attempt was reported in directly reprogramming fibroblasts into osteoblasts by culturing the fibroblasts with a small molecule compound, ALK5 inhibitor II (ALK5 i II), thus solving the problem of tumorigenicity related to the use of retrovirus vectors. A high-quality scaffolding material allowed obtaining a cross-linked nanogel with improved cell adhesion ability. Cross-linked nanogel scaffolds, which are made of polysaccharides, are safer than atelocollagen because, unlike atelocollagen scaffolds, which use animal-derived collagen, they do not contain xenogenic proteins. Moreover, nanogel scaffolds are biodegradable and fully absorbed in the long range after transplantation.

In this study, an application was made of previously developed technology of direct reprogramming by means of a small molecular compound to produce osteoblasts reducing the risks of tumorigenicity related to retrovirus vectors. Moreover, the strategy of a xeno-free scaffold was adopted by using cross-linked nanogels and attempted to improve it with developing more flexible sheet-type morphology as compared to the presently available fiber type. Such an improved morphology is expected to allow an easier and more efficient application including three-dimensional (3D) molding to cope with larger-scale bone defects. The sheet-type polysaccharide structure also enhances the possibility of constructing blocks of living tissue *in vitro* with a shape suitable for transplantation, thus facilitating the transplantation procedure in clinical applications. The results were examined by means of vibrational spectroscopic analyses.

Establishing a method for crafting cross-linked nanogels into sheets of various sizes and thicknesses will open the possibility to develop scaffolds for well plate cultures of various cells and to propose new bone regeneration therapies for large-scale bone defects that are intractable by the presently available technologies and for which no cure has yet been established.

## Experimental Procedures

### Preparation of FD-NanoClip Polysaccharide Scaffolds

Cross-linked nanogel containing pullulan, a natural polysaccharide, was used as the main component of the scaffolding material. The nanogel (substituted with an acryloyl group) was chemically cross-linked with a terminal thiol group polyethylene glycol, molded into a free 3D shape, and freeze-dried to give it a porous structure. Coating with adhesion factor (fibronectin) was applied in order to facilitate the adhesion of osteoblasts and their growth onto the inner parts of the scaffold with three-dimensional orientations (as shown in the following section).

The preparation of the “FD-NanoClip gel” has been described in a previous report (Sato et al., 2018). Briefly, the cholesterol-bearing pullulan (CHP), in which pullulan (average molecular weight = 1 × 10^5^ g/mol) was substituted with 1.2 cholesterol moieties per 100 anhydrous glucoside units, was purchased from NOF Corporation (Tokyo, Japan). Acryloyl group-modified cholesterol-bearing pullulan (CHP-OA) was synthesized using 2-acryloyloxyethyl isocyanate (Showa Denko, Tokyo, Japan). CHP-OA self-assembled into CHP-OA nanogel, which was subsequently cross-linked by means of Michael’s addition of pentaerythritol tetra (mercaptoethyl) polyoxyethylene (PEGSH) (average molecular weight = 1 × 10^4^ g/mol) (NOF CORPORATION) to form NanoClik gel in templates. NanoClik gel was then converted into highly porous NanoClip gel by freezing-induced phase separation. Freezing-induced phase separation of the NanoClik gel yielded a relatively uniform porous structure with a pore diameter of several hundred micrometers (Hashimoto et al., 2015; Hashimoto et al., 2018). To prepare freeze-dried-NanoClip matrix (FD-NanoClip matrix), NanoClip gel was quickly frozen in liquid nitrogen, followed by drying in a vacuum. The pore structures were still observed in FD-NanoClip gel, which contains numerous large pores that were formed by fusion of pre-existing pores in the NanoClip gel through freezing and drying. For fibronectin-coating, NanoClip-FD matrix was soaked in 50 μg/ml fibronectin solution (Wako laboratory chemicals, Osaka, Japan) for 12 h, followed by rinsing in ethanol and drying. The resultant fibronectin-coated NanoClip-FD matrix was hydrated to form fibronectin-coated NanoClip-FD gel.

### Preparation of Fiber-Type Nanogel Polysaccharide Scaffolds

**FIGURE 1:**
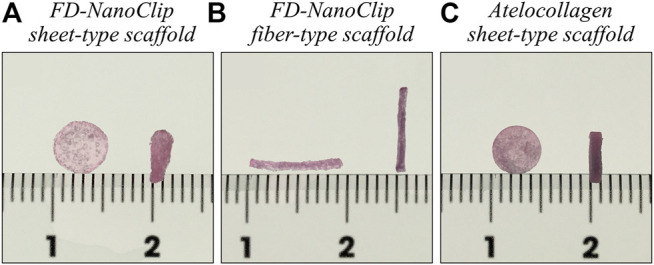
Pictures of **(A)** FD-NanoClip sheet-type, **(B)** FD-NanoClip fiber-type, and **(C)** atelocollagen sheet-type scaffolds used in this study. Each scaffold is in the wet state with a DMEM medium.

The method for producing fiber-type cross-linked nanogel (FD-NanoClip-fiber) scaffold has been described in a previous report (Sato et al., 2018). Exactly, the same procedure for preparation was followed here. The preparation procedure can briefly be described as follows: dried pullulan modified with a cholesteryl group and an acryloyl group (CHP-OA) was dissolved in phosphate-buffered saline (PBS) solution using a magnetic stirrer at 600 rpm for overnight to have a concentration of 30 mg/ml. The cross-linking agent peripheral thiolated polyethylene glycol (PEGSH) was dissolved in PBS solution to reach a concentration of 105 mg/ml. After vortex mixing of the two above solutions in 2:1 balance, the resulting solution was quickly sucked up into Microhematocrit Capillary Tubes with an inner diameter of 1.1 mm (Fisher Scientific) as a mold by the capillary. The gel was then allowed to stand and was incubated at 37°C for 90 min. After incubation time, the fiber-shaped gel was taken out from the mold by pouring PBS through the stump of Microhematocrit Capillary Tubes, washed with PBS, immersed in PBS, and cooled to 4°C. Finally, the fiber-shaped gel was left to stand overnight at −30°C. After thawing the frozen fiber-shaped gel down to room temperature and washing it twice with pure water to eliminate the salt, excess water was drained, and the whole container was frozen into liquid nitrogen and vacuum-dried overnight. The lyophilized gel was immersed in 50 μg/ml fibronectin solution for 12 h and coated with fibronectin. Finally, the porous gel was molded into a fiber having diameter and length of 0.9 and 10 mm, respectively, and then permeated into a 70% ethanol solution for 10 s to remove excess fibronectin. Individual wrapping in sterilized parafilm was performed following sterilization. The packaged gel was again vacuum-dried for 12 h. The completed cross-linked nanogel, as measured in wet state, had a diameter and length of 0.8 and 9.1 mm, respectively (total volume ∼4.6 mm^3^) (referred to as FD-NanoClip fiber, henceforth) (Figure 1B).

### Preparation of Sheet-Type Nanogel Polysaccharide Scaffolds

**FIGURE 2:**
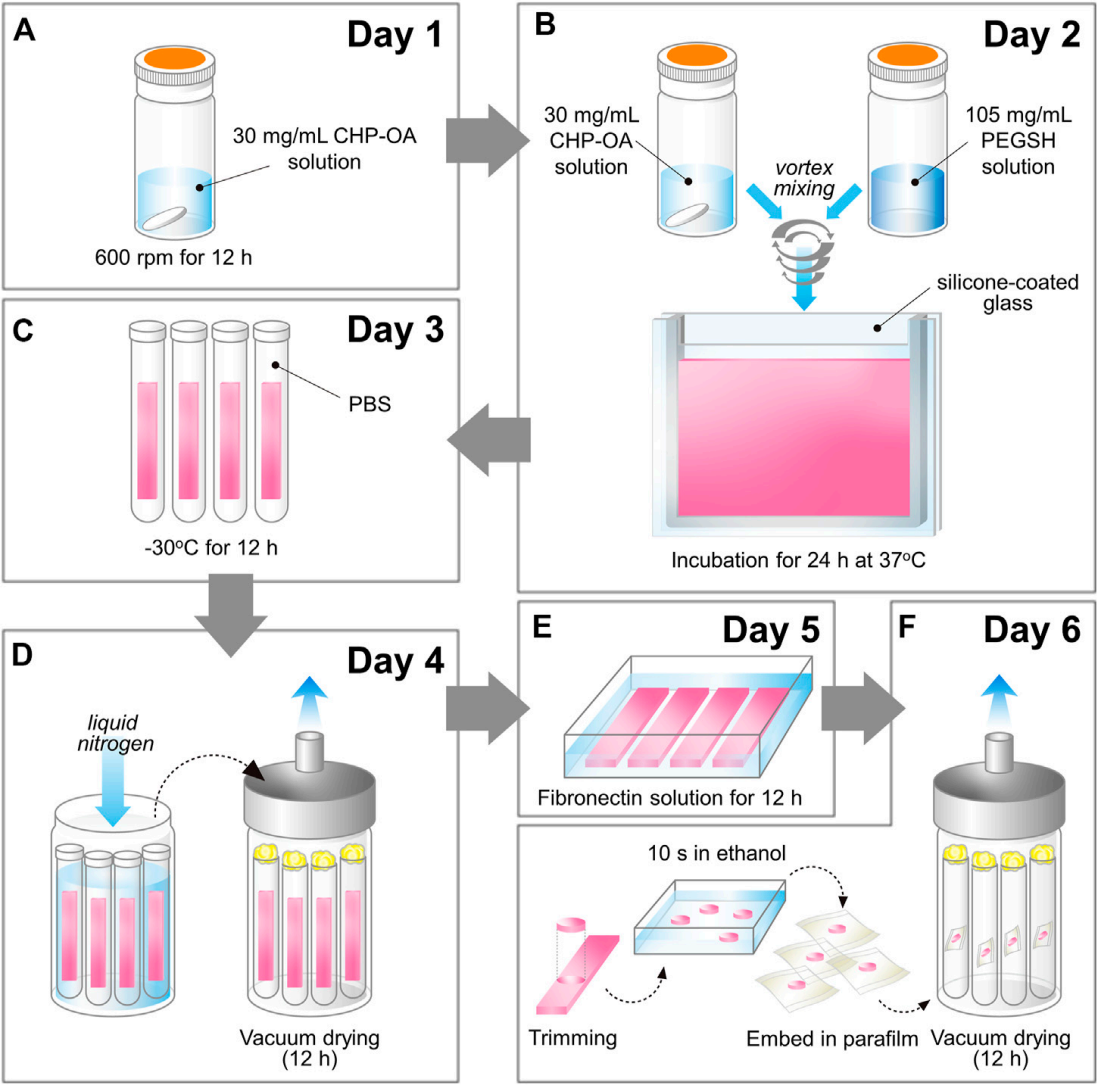
Schematic draft of the method followed in preparing the FD-NanoClip sheet-type scaffold (cf. description in text).

The new method for preparing the sheet-type cross-linked nanogel (FD-NanoClip-sheet) is schematically shown in Figure 2. The preparation procedure can briefly be described as follows: dried pullulan modified with a cholesteryl group and an acryloyl group (CHP-OA) was dissolved in phosphate-buffered saline (PBS) solution using a magnetic stirrer at 600 rpm for overnight to have a concentration of 30 mg/ml (Figure 2A). The cross-linking agent peripheral thiolated polyethylene glycol (PEGSH) was dissolved in PBS solution to reach a concentration of 105 mg/ml. After vortex mixing of the two above solutions in 2:1 balance, the resulting solution was quickly poured into a mold made of a silicone-coated glass plate with a 1.5 mm spacer. The gel was then allowed to stand and was incubated at 37°C for 24 h (Figure 2B). After incubation time, the sheet-shaped gel was taken out from the mold, cut into strips, washed with PBS, immersed in PBS, and cooled to 4°C. Finally, the stripes were left to stand overnight at −30°C (Figure 2C). Note that preliminary cutting into strips allowed reducing cracking due to condensation during freeze-drying. After thawing the frozen gel stripes down to room temperature and washing them twice with pure water to eliminate the salt, excess water was drained, and the whole container was frozen into liquid nitrogen and vacuum-dried overnight (Figure 2D). The lyophilized gel was immersed in 50 μg/ml fibronectin solution for 12 h and coated with fibronectin (Figure 2E). Finally, the porous gel was molded into a disk sheet having a diameter and thickness of 6 and 1.2 mm, respectively, and then permeated into a 70% ethanol solution for 10 s to remove excess fibronectin. Individual wrapping in sterilized parafilm was performed following sterilization. The packaged gel was again vacuum-dried for 12 h. The completed disk sheet cross-linked nanogel as measured in the wet state had a diameter and thickness of 5 and 1.0 mm, respectively (total volume ∼19.6 mm^3^) (referred to as FD-NanoClip sheet, henceforth) (Figure 2F) (Figure 1A).

### Preparation of Sheet-Type Atelocollagen Scaffolds

For comparison, a columnar collagen sponge (AteloCell^®^ Atelocollagen sponge MIGHTY, KOKEN, Tokyo) with diameter and thickness of 5 and 3 mm, respectively, was used, which was accurately molded into sheets with a thickness of 1.0 mm and diameter of 5 mm using a microtome blade. The completed disk sheet collagen sponge as measured in the wet state had a diameter and thickness of 5 and 1.0 mm, respectively (total volume ∼19.6 mm^3^) (referred to as atelocollagen sheet, henceforth) (Figure 1C).

FD-NanoClip sheets and atelocollagen sheets had approximately the same volume in the wet state. According to volume calculations in the wet state, five pieces of FD-NanoClip fiber had approximately the same volume as one piece of FD-NanoClip sheet or atelocollagen sheet (22.8 vs. 19.6 mm^3^).

### Conversion of Human Fibroblasts into Osteoblasts on 3D Scaffold

HDFs derived from the breast of a 18-years-old male were purchased from Toyobo Life Science (Osaka, Japan) and cultured in Dulbecco’s minimum essential medium (DMEM) (Nacalai Tesque, Kyoto, Japan) supplemented with 100 mM non-essential amino acids, 100 U/ml penicillin 100 μg/ml streptomycin, and 10% fetal bovine serum (FBS) in 5% CO_2_/95% humidified air at 37°C (complete culture medium). The medium was refreshed every 3–4 days. ALK5 inhibitor II (ALK5 i II) and 1α, 25-dihydroxy Vitamin D3 (VitD3) were purchased from StemRD (ALK-010) and Cayman (Cat. no. 71820). The osteogenic medium (OM) consisted of the complete medium supplemented with 50 μg/ml ascorbic acid, 10 mM β-glycerol phosphate, and 100 nM dexamethasone. HDFs (15 μL of 1.5 × 10^5^ cell-containing medium) were seeded on FD-NanoClip fiber, FD-NanoClip sheet, or atelocollagen sheet. In order to allow for a quantitative comparison of the degree of calcification, both the number of cells seeded in each scaffold and the volume of the scaffolds were matched. Each scaffold made of FD-NanoClip sheet and atelocollagen sheet (having the same volume) was placed in a separate well. On the other hand, five pieces of FD-NanoClip fiber, whose cumulative volume was almost the same as that of one of the above sheets, were placed side by side in each well. Each cell-filled scaffold was incubated for 2 h to promote the adhesion of cells onto the scaffolds. After 2 h, the cell-filled scaffolds were transferred into 12-well plates and cultured in a complete medium for 24 h. Then, the culture medium was replaced by OM supplemented with 4 μM of ALK5 i II and 5 nM VitD3 to induce conversion into osteoblasts. In this way, dOBs were directly converted from HDFs during the culture on 3D scaffolds, thus FD-NanoClip sheet-dOB, FD-NanoClip fiber-dOB, and atelocollagen sheet-dOB complexes were successfully formed. The culture medium was refreshed every 3–4 days.

### Alizarin Red S Staining Method

To confirm whether osteoblast-like cells formed a calcified substrate inside the three-dimensionally cultured scaffold, FD-NanoClip sheet-dOB, FD-NanoClip fiber-dOB, and atelocollagen sheet-dOB complexes were fixed with 95% ethanol on day 21. Then, staining was performed with Alizarin Red S solution (Sigma Aldrich). Calcium is chelated by alizarin red S in an aqueous solution, forming a complex. The unbound calcium stain was carefully removed through several changes of D.I. H_2_O. Successively, the stained complexes were transferred to 12 different wells, in which 1,000 μl/well of 10% formic acid (FUJIFILM Wako Pure Chemical Corporation, Osaka, Japan) were added. Each sample was dissolved upon shaking, and the supernatant was collected after 15 min. The collected supernatant was measured for absorbance at 405 nm using a spectrometer (McNeill et al., 2020), and the amount of calcification was quantitatively compared with sampling *n* = 3.

### Real-Time Reverse Transcription Polymerase Chain Reaction

FD-NanoClip sheet-dOB, FD-NanoClip fiber-dOB, and atelocollagen sheet-dOB complexes were harvested on day 14, lysed with ISOGEN II (Nippon Gene, Tokyo, Japan) (homogenized with BioMasher II; Nippi, Tokyo, Japan), and extracted of their contained RNA. The extracted RNA was reverse-transcribed into cDNA using ReverTraAce qPCR RT Master Mix (TOYOBO). Then, real-time RT-PCR was performed to evaluate expression levels of mRNA for alkaline phosphatase and osteocalcin genes using real-time PCR Master Mix (Applied Biosystems, Waltham, MA, United States) and matching probes and primers (Thermo Fisher Science) on a Step One Plus Real-Time PCR System (Applied Biosystems). All values (average ±SD) were normalized with respect to the GAPDH mRNA level in each sample and expressed relative values (*n* = 4). The primer and probes used this experiment are listed in Table 1.

**TABLE 1:** The primer/probes used for real-time RT-PCR.

Gene name	Sequence/product number
GAPDH	Forward primer: 5′-CTC​AAG​ATC​ATC​AGC​AAT​GCC​TC-3′
—	Reverse primer: 5′-CCC​ACA​GCC​TTG​GCA​GC-3′
—	Probe: 5′-CGTGATGGCCGCGG-3′
Alkaline phosphatase	Forward primer: 5′-TGA​CAC​CTG​GAA​GAG​CTT​CAA​A-3′
—	Reverse primer: 5′-CCG​TGC​GGT​TCC​AGA​TG-3′
—	Probe: 5′-AGA​TAC​AAG​CAC​TCC​CAC-3′
Osteocalcin	Hs01587814_g1 BGLAP (Thermo Fisher Scientific)

### Vibrational Spectroscopy Assessments

Raman analyses were conducted on the three types of scaffold investigated, in order to assess their structural differences at the molecular scale. Spectra were collected on glucan polysaccharides and atelocollagen samples by means of a LabRAM HR800 (Horiba/Jobin Ivon, Kyoto, Japan) operated in microscopic measurement mode with confocal imaging capability in two dimensions. The light source was a HeNe laser operating at 633 nm with a power of 10 mW. A holographic notch filter was incorporated into the system to enable the acquisition of Raman spectra with conditions optimized by the manufacturer for high sensitivity. The Raman scattered light was monitored by a single monochromator connected with an air-cooled charge-coupled device (CCD) detector (Andor DV420-OE322; 1,024 × 256 pixels). The acquisition time was fixed at 10 s. Spectral deconvolution into Lorentzian bands was made by means of commercially available software (Origin 9.1, OriginLab Co., Northampton, MA, United States). An average of 20 Raman measurements per scaffold was collected at different random locations and average spectra were analyzed.

Attenuated total reflectance Fourier transform infrared (ATR-FTIR) spectra were recorded using a high sensitivity spectroscope (Spectrum 100FT-IR Spotlight 400; PerkinElmer Inc., Waltham, MA, United States). The spectral resolution of this equipment was 0.4 cm^−1^. Average ATR-FTIR spectra were computed using six independent measurements performed on *n* = 3 samples. Spectral acquisition and preprocessing of raw data included baseline subtraction, smoothing, and normalization, which were carried out using commercially available software (Origin 8.5, OriginLab Co., Northampton, MA, United States). On days 14 and 21 of cultures, we performed ATR-FTIR spectroscopic analyses to evaluate the quality of the bone matrix produced in tissue regenerated by FD-NanoClip-dOB and atelocollagen-dOB complexes. The samples used for ATR-FTIR did not require any manipulation or preparation.

### Statistical Analyses

Data were expressed as mean ± standard deviation. Statistical significance was analyzed using the Tukey-Kramer test; *p* < 0.05 was taken as the threshold condition for significance and assigned one asterisk label. Two asterisks were instead assigned to *p* < 0.01.

## Experimental Results

### Raman Spectra From Polysaccharides and Atelocollagen Scaffolds

**FIGURE 3:**
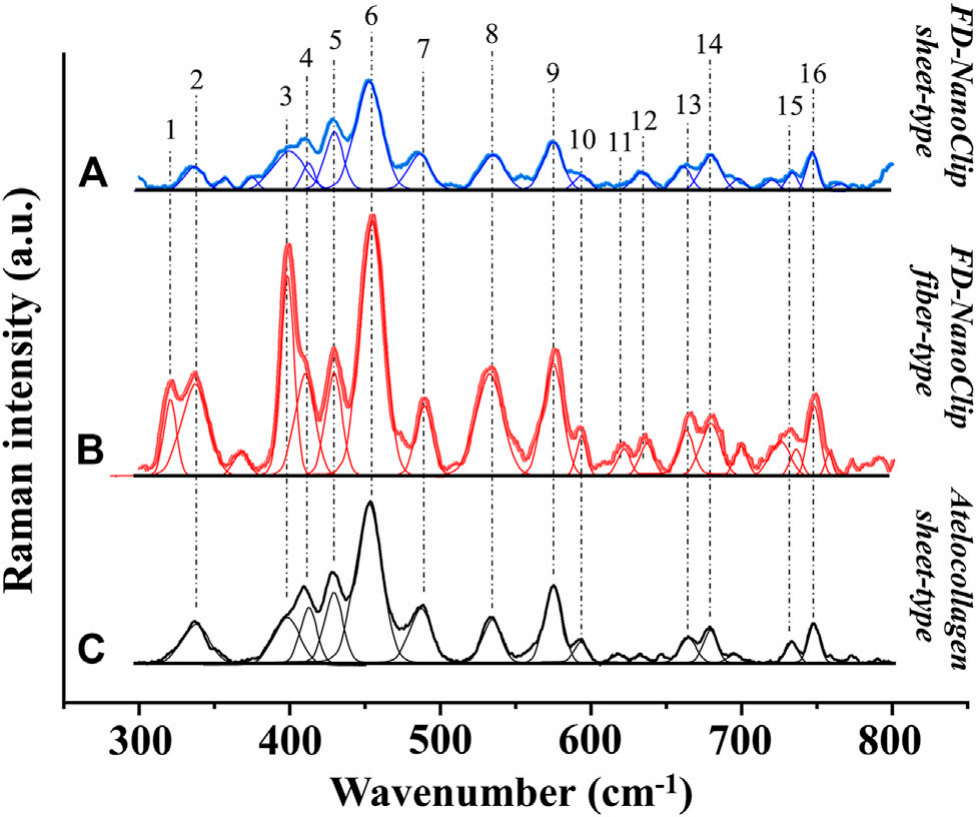
Deconvoluted Raman spectra of **(A)** FD-NanoClip sheet-type, **(B)** FD-NanoClip fiber-type, and **(C)** atelocollagen sheet-type scaffolds in the frequency interval 300–800 cm^−1^; bands are labeled and vibrational origins assigned as shown in Table 2.

FD-NanoClip sheet-type, FD-NanoClip fiber-type, and atelocollagen sheet-type scaffolds are shown in Figures 1A–C, respectively. All scaffolds presented highly wrinkled and porous structures with similar morphologies. The fibrous polysaccharide scaffold was previously reported as hierarchically structured with respect to its porosity with a bimodal pore distribution (Sato et al., 2018; Hashimoto et al., 2018; Hashimoto et al., 2015). A quite similar structure could be obtained in the sheet-type polysaccharide structure. The sheet-type polysaccharide scaffold was hierarchically structured with respect to its porosity with a bimodal pore distribution. Similarly, the spongy atelocollagen scaffold possessed a high degree of large porosity and pore interconnectivity designed to promote tissue ingrowth (Izadifar et al., 2012). Raman spectra were systematically collected on all the as-fabricated investigated scaffolds. Comparisons were then carried out to study their structural and chemical characteristics at the molecular scale. Deconvoluted Raman spectra of FD-NanoClip sheet-type, FD-NanoClip fiber-type, and atelocollagen sheet-type scaffolds in the frequency interval 300–800 cm^−1^ are shown in Figures 3A–C, respectively. The 16 main bands of the collected spectra were labeled and their vibrational origins assigned, as shown in Table 2 (Smith et al., 1969; Diem et al., 1984; Zhbankov et al., 1997; Wiercigroch et al., 2017). The presence of different saccharides in the nanogel scaffolds was assessed based on a previously presented analytical algorithm, which refers to a database of Raman spectra from pure molecules (Horiguchi et al., 2019).

**TABLE 2:** Main deconvoluted band components of the Raman spectra labeled in Figure 3 with their most recent vibrational assignments on the same material. The original assignments can be found in the bibliography section of these previous documents.

Label	cm^−1^	Assignments	References
1	320	Triple-helical polymer (Pro-Pro-Gly)_10_ (only atelocollagen)	Sato et al. (2018), Yamamoto et al. (2018)
2	344	Endocyclic C-C-O bending in D-(+)-maltose monohydrate	Hashimoto et al. (2015)
3	398	Triple-helical polymer (Pro-Pro-Gly)_10_ (only atelocollagen)	Sato et al. (2018), Yamamoto et al. (2018)
4	403	Fructosyl C-C-O deformation in sucrose and dextran	Hashimoto et al. (2015), Hashimoto et al. (2018)
5	428	Ring C-C-O bending in D-(+)-maltose monohydrate	Hashimoto et al. (2015)
6	452	Glucosyl C-C-O bending in D-(+)-maltose monohydrate	Hashimoto et al. (2015)
7	489	C-C-C in-plane bending in pullulan	Hashimoto et al. (2018)
8	540	Glucose C-C and C-O stretching in sucrose and dextrose	Hashimoto et al. (2015)
9	575	C-C-O bending in sucrose (fructosyl) and pullulan	Hashimoto et al. (2015), Hashimoto et al. (2018)
10	593	Fructosyl C-C-O bending in sucrose	Hashimoto et al. (2015)
11	621	O-C-O bending in dextran	Hashimoto et al. (2015), Hashimoto et al. (2018)
12	633	Ring deformation in sucrose (fructosyl) and maltose (glucosyl)	Hashimoto et al. (2015)
13	664	C-C-O bending in pullulan	Hashimoto et al. (2015), Hashimoto et al. (2018)
14	680	Ring C-O stretching and C-O-C bending in α-methyl-D-mannoside	Hashimoto et al. (2015), Hashimoto et al. (2018)
15	730	Fructosyl C-C-O bending in sucrose	Hashimoto et al. (2015)
16	748	CH_2_ rocking in D-(+)-raffinose pentahydrate	Hashimoto et al. (2015)

**FIGURE 4:**
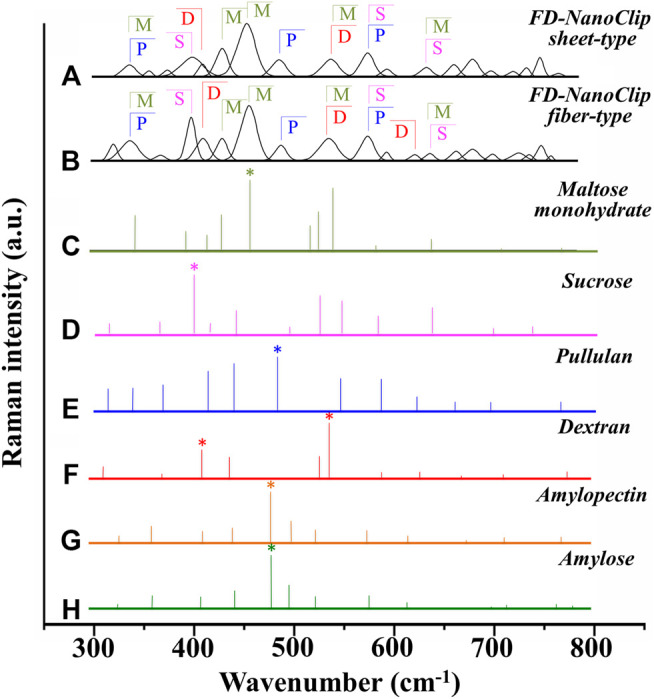
Deconvoluted bands from FD-NanoClip scaffolds (sheet- and fiber-type in **(A)** and **(B)**, respectively) and normalized diagrams from spectra of selected elementary saccharide molecules [**(C–H)**; cf. labels]. Asterisks indicate fingerprint bands for different saccharides. The abbreviations M, S, D, and P stand for D-(+)-maltose monohydrate, D-(+)-sucrose, dextran, and pullulan, respectively.

Deconvoluted bands from the scaffolds and normalized diagrams from spectra of selected elementary saccharide molecules are shown in Figure 4. As seen, the spectra of the two polysaccharide scaffolds were interpreted according to the vibrational features of disaccharides (D-(+)-sucrose and D-(+)-maltose monohydrate) and α-D-glucose polymers (pullulan, dextran, amylose, and amylopectin). α-D-glucose polymers present relevant Raman signals in the low-frequency region and are characterized by the presence of their main bands in the frequency interval 470–480 cm^−1^ and at ∼540 cm^−1^ for amylose/amylopectin and dextran, respectively (Zhbankov et al., 1997). On the other hand, the absence of the latter band is a distinctive characteristic of pullulan. The reason for this difference resides in the character of the bond at the C4 location. Volumetric substitution of the OH group at the C4 location leads to the appearance of an intense band at ∼480 cm^−1^, while no substitution is characterized by the presence of the Raman band at ∼540 cm^−1^. The former is the case of amylose and amylopectin, in which the glucoside bond ClOC4 is formed, while the latter is the case of dextran, in which the structure of the OH group at C4 is not substituted and ClOC6 bonds are found between the links. In the pullulan macromolecule, both ClC4 and ClC6 bonds (with a prevalence of C1C4 bonds) can be found at the elementary links. Accordingly, in the Raman spectrum of this polymer, the band at ∼480 cm^−1^, which represents the C1C4 bond, is the most pronounced (cf. diagram in Figure 4). Upon translating these notions into the analyses of the present polysaccharide scaffolds, it appears that both FD-NanoClip scaffolds, independent of morphology, included substantial fractions of pullulan and dextran, but no significant amounts of amylose or amylopectin. However, explaining the main band and another prominent band observed in the spectrum of both FD-NanoClip scaffolds (i.e., the bands located at ∼452 and ∼402 cm^−1^, respectively) required also considering the presence of disaccharides. The band at 452 cm^−1^, which is assigned to bending mode of the glucosyl CCO group (Wiercigroch et al., 2017) can be considered a fingerprint for D-(+)-maltose monohydrate. On the other hand, the band at 403 cm^−1^, which is assigned to the deformation mode of the endocyclic CCO group in the fructosyl moiety (Wiercigroch et al., 2017) is a fingerprint for sucrose with its spectral position being characteristic for the α-anomer (Watson and Crick, 1953). An additional fingerprint from D-(+)-maltose monohydrate with the same vibrational origin of the band at 452 cm^−1^ was found at ∼428 cm^−1^. Additional bands from this disaccharide were found at ∼344 cm^−1^ (bending mode of the endocyclic CCO group) (Wiercigroch et al., 2017) and at ∼540 cm^−1^, in overlap with bands from pullulan and dextran, respectively. A comparison between the spectra of sheet-type and fiber-type FD-NanoClip scaffolds suggests the presence of similar saccharide structures but also a larger fraction of sucrose in the latter scaffold (cf. intensities of the band at 403 cm^−1^ in Figures 4A,B). Both FD-NanoClip fiber- and sheet-type scaffolds might also contain a fraction of dextran as suggested by the fingerprint band of this disaccharide at ∼403 cm^−1^ (bending mode of the CCO group in the fructosyl unit) (Wiercigroch et al., 2017) and the band component at ∼540 cm^−1^ (in overlap with a vibration from maltose monohydrate).

Despite the morphological similarity between the low-frequency spectra of polysaccharides and atelocollagen scaffolds, some of the Raman bands in these spectra have different vibrational origins. As a matter of fact, the atelocollagen scaffold consists of both proteins and polysaccharides. A complete description of the Raman spectrum of the atelocollagen scaffold has been given in a previously published paper (Horiguchi et al., 2019). Briefly, the main band seen at ∼450 cm^−1^ has the same origin described above for the polysaccharide scaffolds (bending mode of the glucosyl CCO group) (Wiercigroch et al., 2017). Similar reasoning holds for the band at ∼430 cm^−1^ with the same vibrational origin. On the other hand, the broad and relatively weak signal at ∼400 cm^−1^ (Figure 3C) is assigned to the (Pro-Pro-Gly)_10_ triple-helical polymer (Smith et al., 1969; Diem et al., 1984). Additional contributions to the spectrum of atelocollagen include torsional modes of polypeptide backbone from α-helical polypeptides, triple-helical polymer (Pro-Pro-Gly)_10_, and protein collagen. An important difference in the low-frequency region between the spectra of saccharides and atelocollagen scaffolds is the relatively sharp band at ∼320 cm^−1^ seen only in the spectrum of the latter. Also, this band can be assigned to triple-helical polymer (Pro-Pro-Gly)_10_ and was also found in glycylproline (Smith et al., 1969; Diem et al., 1984).

In summary, the Raman spectroscopic characterizations of different scaffolds revealed that differences between FD-NanoClip fiber- and sheet-type scaffolds not only were of morphological nature but also covered subtle but important features of chemical nature: the former scaffold being richer in sucrose and dextran.

### Cell Conversion on FD-NanoClip and Atelocollagen Scaffolds

**FIGURE 5:**
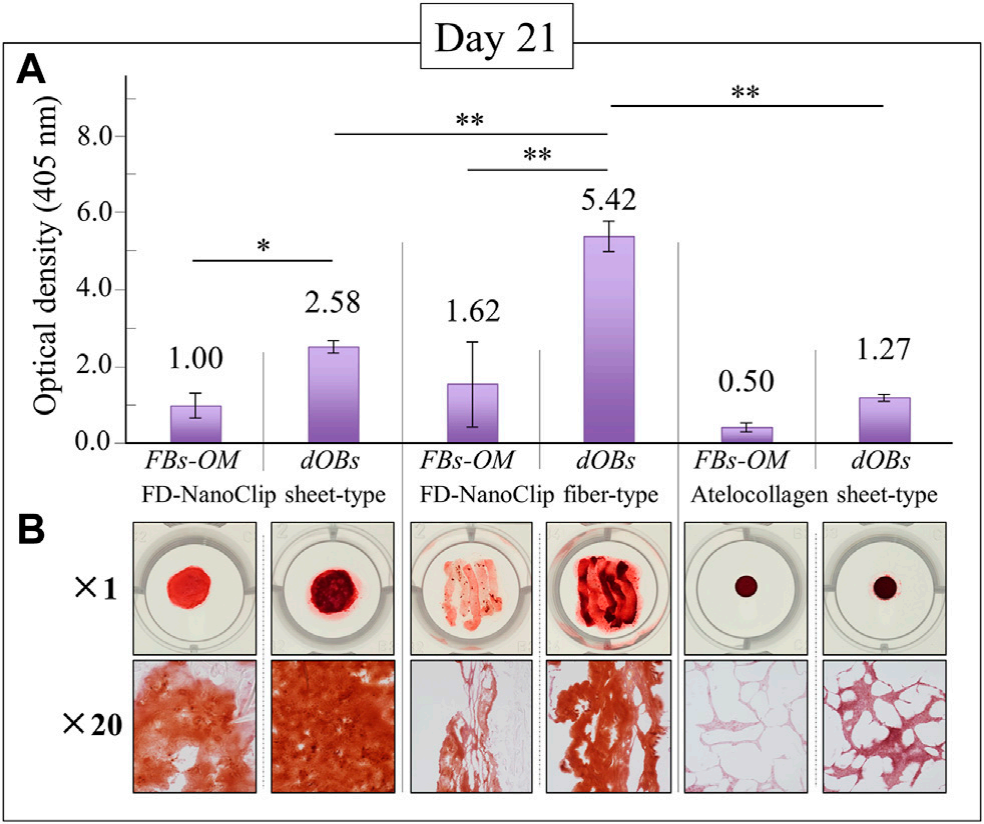
Results of the Alizarin Red S staining method at day 21 for FD-NanoClip sheet-dOB, FD-NanoClip fiber-dOB, atelocollagen sheet-dOB complexes, and FBs-OM (fibroblasts cultured with osteogenic medium) (cf. labels); quantitative plots of the degree of calcification with statistical validation (*n* = 3) and related optical pictures in the **(A)** and **(B)** parts of the figure, respectively. Average values are explicitly indicated on top of each plot. One and two asterisks refer to *p* < 0.05 and *p* < 0.01, respectively.

The results of calcification by Alizarin Red S staining on the FD-NanoClip sheet-dOB, FD-NanoClip fiber-dOB, and atelocollagen sheet-dOB complexes at day 21 are shown in Figure 5. In all scaffolds, the dOB group was deeply stained in red as compared to the control group, indicating that calcification was greatly enhanced by the cell conversion process (cf. optical pictures in the lower part of Figure 5). The degree of calcification was evaluated as follows. Scaffolds were dissolved in formic acid by shaking them for 15 min. The supernatant was harvested and absorbance at 405 nm was measured (*n* = 3) (cf. plot in the upper part of Figure 5).

The results show that all scaffolds reached a degree of calcification higher than that of the undifferentiated control group (with statistical significance). Both FD-NanoClip sheet-dOB and FD-NanoClip fiber-dOB complexes enabled a degree of calcification higher than that reached in the sheet-type atelocollagen scaffold for exactly the same culture conditions. However, the FD-NanoClip fiber-type scaffold enabled a degree of calcification more than twice higher than that of the NanoClip sheet-type one. Note that the atelocollagen sheet-dOB complex underperformed in the calcification process with respect to the FD-NanoClip sheet-dOB complex with the same morphology, thus indicating a better intrinsic stimulus for the differentiated cells toward calcification on polysaccharides as compared to atelocollagen. The reason why the control group is dyed red in all scaffolds is believed to be that the control group was cultured in an osteogenic medium, resulting in a small amount of calcification. Furthermore, it is possible that the 3D culture promoted calcification. Our previous report (Sato et al., 2018) showed that alizarin red S staining of fiber-type FD-NanoClip without HDF cultured in bone-forming medium did not stain red.

FD-NanoClip-dOB complexes with different morphologies and the atelocollagen-dOB complex were formed at day 14 of culture in an osteogenic medium. The complexes were harvested and their RNA extracted in order to quantify osteoblast differentiation markers by means of real-time RT-PCR.

**FIGURE 6:**
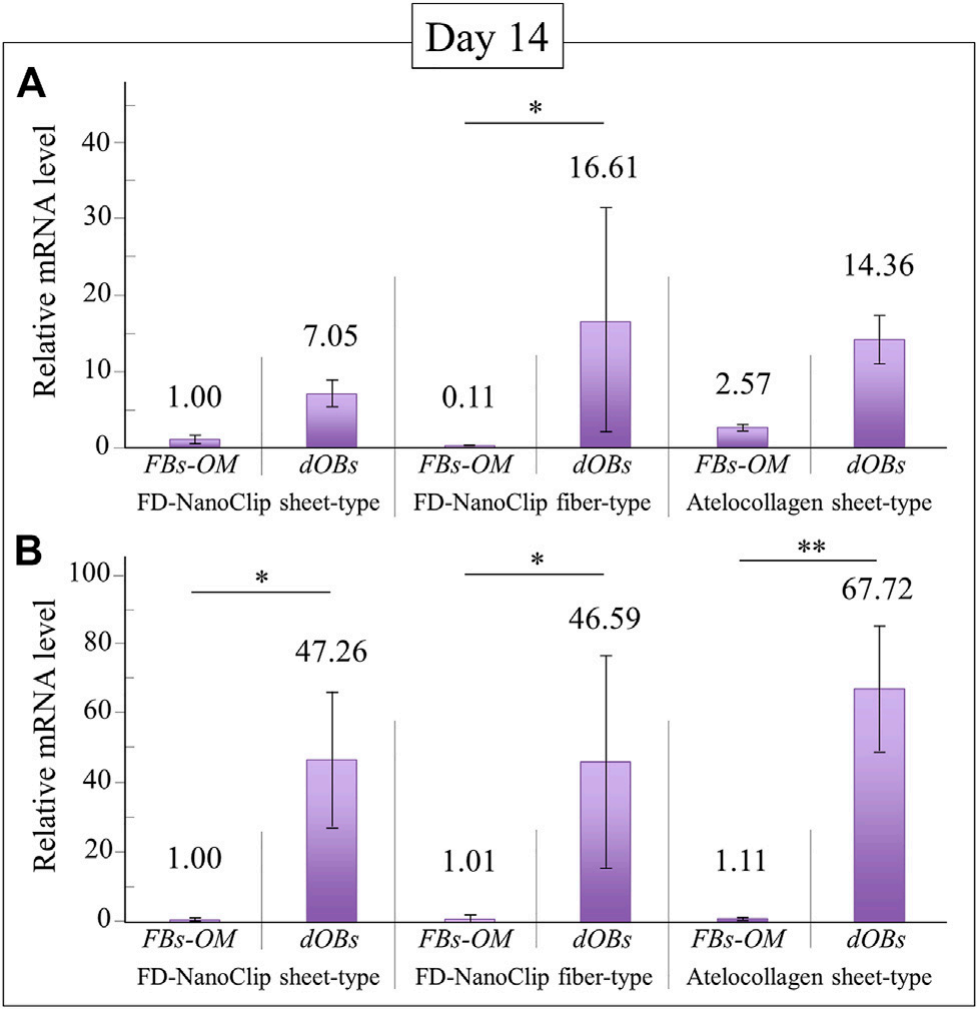
Quantitative results of **(A)** alkaline phosphatase and **(B)** osteocalcin from real-time RT-PCR analyses at day 14 for FD-NanoClip sheet-dOB, FD-NanoClip fiber-dOB, atelocollagen sheet-dOB complexes, and FBs-OM (fibroblasts cultured with osteogenic medium) (cf. labels). The primer probes used this experiment are listed in Table 1. Average values are explicitly indicated on top of each plot (*n* = 4). One and two asterisks refer to *p* < 0.05 and *p* < 0.01, respectively.

Figures 6A,B show quantitative results of alkaline phosphatase and osteocalcin, respectively. Independent of the expression index selected, all complexes reached a degree of calcification higher than the undifferentiated control group although with different degrees of statistical significance (cf. labels in inset). Regarding the plot of alkaline phosphatase (Figure 6A), the FD-NanoClip fiber-dOB complex showed a value ∼2.3 times higher than that of FD-NanoClip sheet-dOB complex and comparable with that of the atelocollagen-dOB complex (although with a higher statistical scatter). Conversely, the quantitative results on the expression of osteocalcin revealed the highest level for the atelocollagen-dOB complex and comparable levels for the two morphologically different types of FD-NanoClip-dOB complexes (Figure 6B). However, the scatter in the results (*n* = 4) was high and the detected differences were not substantiated by statistical significance (cf. labels in inset). It should also be noted that FD-NanoClip allowed the cells to express osteogenic markers at levels as high as those of atelocollagen.

### Attenuated Total Reflectance Fourier Transform Infrared Analyses of Bone Tissue Grown Within the Scaffolds

**FIGURE 7:**
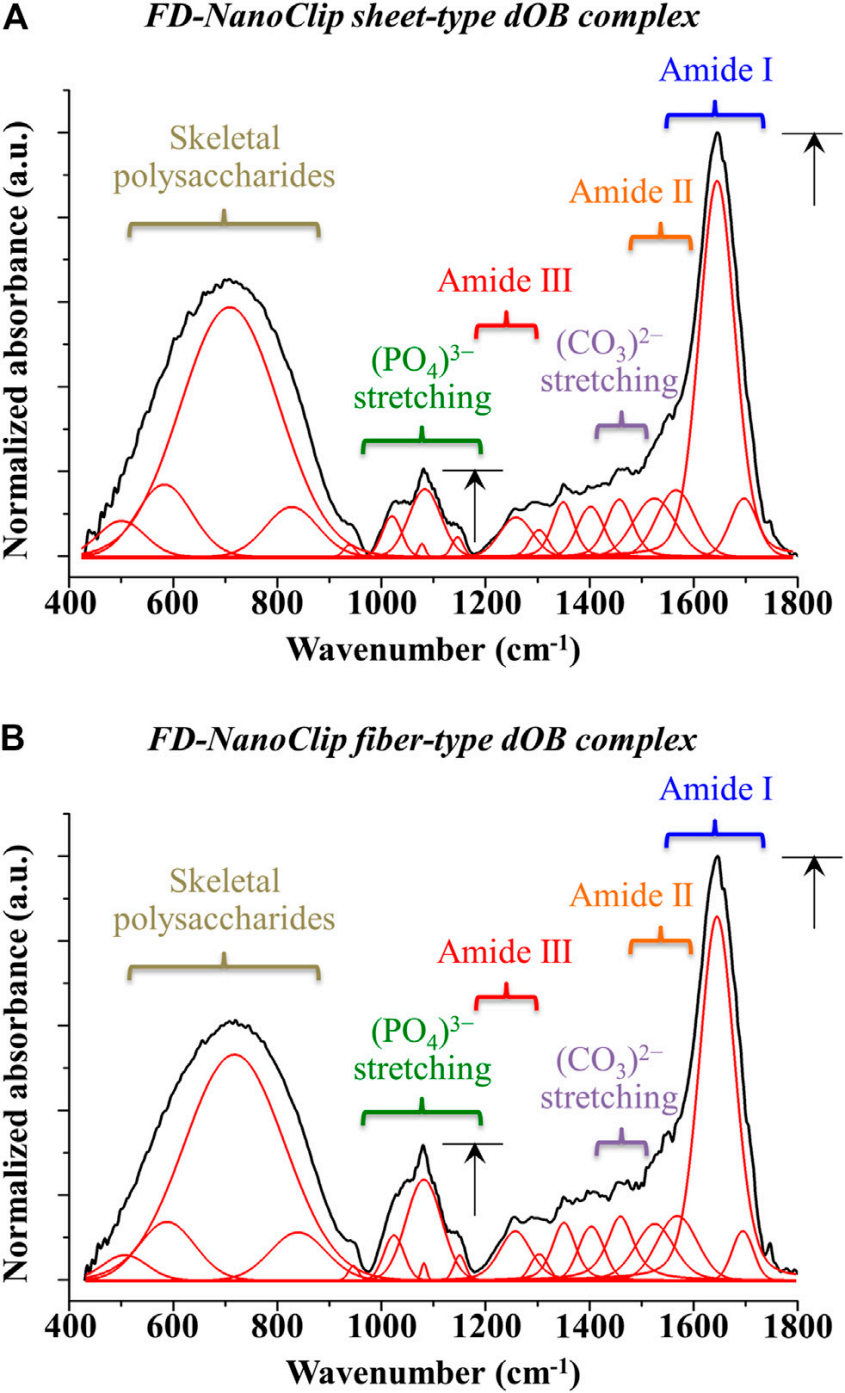
ATR-FTIR spectra of **(A)** FD-NanoClip sheet-dOB and **(B)** FD-NanoClip fiber-dOB complexes at day 21 in the frequency interval 400–1800 cm^−1^; spectra are normalized to the Amide I band centered at around 1700 cm^−1^. Assignments are given with labels in inset.

In order to clarify the differences recorded between FD-NanoClip-dOB complexes in quantitative assessments of osteoblast differentiation markers for the cases of fiber- and sheet-type scaffolds (cf. previous section), the ATR-FTIR spectra of the two complexes were examined and compared at day 21. The spectra are shown in Figures 7A,B for sheet and fiber scaffold morphologies, respectively. The spectra were normalized to the Amide I band, which was the most pronounced band in the examined spectral zone and appeared in the frequency interval between 1,600 and 1800 cm^−1^ (cf. labels in inset to Figure 7). Leaving aside signals from the skeletal vibrations of polysaccharide scaffolds, with both scaffolds commonly presented in the interval 400–900 cm^−1^ (already discussed in the context of Raman characterizations), stretching vibrations from the inorganic apatite phase of bone tissue were seen between 1,000 and 1,200 cm^−1^. Additional signals from bone proteins referred to as Amide II and Amide III were found at 1,500–1,600 cm^−1^ and 1,200–1,300 cm^−1^, respectively. The calcium carbonate structure of bony apatite was detected at ∼1,460 cm^−1^. The intense signals from both the mineral fraction of hydroxyapatite and the three amides confirmed the formation of significant amounts of bone tissue in both types of scaffold (Lebon et al., 2016). However, the FD-NanoClip fiber-dOB scaffold contained a higher fraction of mineral hydroxyapatite. The ratio between the intensity of the apatite (PO_4_)^3-^ band centered at ∼1,050 cm^−1^ and that of the protein Amide I band centered at ∼1,700 cm^−1^ are usually referred to as the mineral-to-matrix ratio (Boskey and Mendelsohn, 2005). This ratio, which is considered as a measure of the bone turnover rate and osteoid formation (Isaksson et al., 2010) was computed from the spectra in Figure 7 as 0.2 and 0.32 for the FD-NanoClip sheet-dOB and FD-NanoClip fiber-dOB complexes, respectively (cf. black arrows in inset to Figure 7). The ∼30% higher mineral-to-matrix ratio in the fiber-type scaffold reflects a higher degree of mineralization of the bone tissue as compared to the sheet-type scaffold, which is in agreement with the calcification data given in Figure 5. Another parameter of bone quality is the so-called carbonate-to-phosphate ratio (Gourion-Arsiquaud et al., 2009; Ren et al., 2014), which was determined from the ratio between the band at ∼1,450 cm^−1^ (associated with stretching of the (CO_3_)^2−^ unit) and the band at ∼1,050 cm^−1^ (associated with stretching of the (PO_4_)^3−^ unit). Note that the carbonate band at ∼850 cm^−1^ could not be used, because this band was embedded into a spectral region dominated by the skeletal vibrations from polysaccharides belonging to the scaffolds. The measured ratio was ∼1.0 and 0.7 for the FD-NanoClip sheet-dOB and FD-NanoClip fiber-dOB complexes, respectively. From a crystallographic viewpoint, both values of the carbonate-to-phosphate ratio are high and should be considered as representative of marked off-stoichiometric conditions of the bone mineral (Isaksson et al., 2010). Finally, ATR-FTIR characterizations were also conducted on the atelocollagen sheet-dOB complex (not shown) and found mineral-to-matrix and carbonate-to-phosphate ratios equal to 0.14 and 1.2, respectively. However, the former parameter cannot directly be compared to those found for polysaccharide scaffolds because the atelocollagen scaffold itself is made of collagen. Therefore, the mineral-to-matrix ratio determined by ATR-FTIR for this scaffold is likely underestimated. On the other hand, the high value found for the carbonate-to-phosphate ratio indicates a highly defective structure for the bone tissue grown by the dOBs on the collagen scaffold.

## Discussion

Research on bone regeneration has a long history, and a number of different approaches have been taken to accomplish its effectiveness so far, such as the use of biomaterial scaffolds (Tang et al., 2021), growth factors (Oliveira et al., 2021), gene therapies (Leng et al., 2020), and local transplantation of osteogenic cells (Hutchings et al., 2020). Auto-bone grafts or allo-bone grafts have been used as the standard for bone repair; however, they have considerable limitations and problems, including donor site injury, graft resorption, high cost, scarcity of graft, and disease transmission. Thus, nowadays the most established and regular methods for bone repair consist in applying artificial biomaterial scaffolds on site for their convenience. Although they do not have the osteoinductive ability by themselves, they can enhance bone regeneration by their osteoconductive ability. Atelocollagen (Teruplug®; Olympus Terumo Biomaterials Co., Tokyo, Japan) (Chang and Cheng, 2021), hydroxyapatite (HA: NEOBONE®; Covalent Materials Co., Tokyo, Japan) (Sasaki et al., 2018), tricalcium phosphate (beta-TCP: OSferion®; Olympus Co., Tokyo, Japan) (Kaneuji et al., 2021), and biphasic calcium phosphate (HA + beta-TCP: Triosite^TM^; Zimmer, Bretagne, France) (Hooper et al., 2013) have been used in clinical practice. Moreover, there are a number of clinical studies accomplishing bone regeneration on site by transplanting mesenchymal stem cells together with these biomaterial scaffolds (Wang et al., 2013; Liu et al., 2014; Jin and Lee, 2018). However, gaining bone regeneration at large bone defects parts is still challenging. Therefore, recently, genes and/or RNAi modified osteoinductive artificial scaffolds (Laird et al., 2021; Sun et al., 2021) and genetically arranged high functional cell seeded scaffolds (Sato et al., 2018; Gugliandolo et al., 2021; Jiang et al., 2021) have been considered as new upcoming bone regeneration strategies.

This study investigated the osteoinductive ability of small molecule-driven osteogenic cells with three types of biomaterial scaffolds and proved that both polysaccharide and atelocollagen scaffolds have biologically active effects. However, it also indicates that the structure and the stoichiometry of the bony tissue grown by cells on different scaffolds could greatly differ according to both morphology and chemical structure of the scaffold. Such differences were clear even among similar polysaccharide scaffolds presenting subtle chemical differences. The recorded differences in the degree of calcification and in alkaline phosphatase expression index between sheet- and fiber-type polysaccharide scaffolds are consistent with each other (2.1–2.3 times larger in the latter type) and could be related to differences in surface area per unit volume, SV. As a matter of fact, the two types of scaffold were tested for the same volume but, given their different morphologies, they greatly differed in SV value (∼5.5 larger in the fiber-type scaffold). Note that no difference was recorded in osteocalcin expression index between polysaccharide scaffolds with different morphologies. This suggests that, despite the similar stimulus received by cells for producing calcified tissue (i.e., a similar osteocalcin expression index), different degrees of calcification were reached. The lower efficiency of the calcification process for polysaccharide scaffolds with sheet-like morphology could be a consequence of its lower SV value. However, the detection of lower quality parameters for the bone tissue could also relate to chemical factors affecting the osteogenic activity of dOBs or be intrinsic to dOBs themselves.

Vibrational spectroscopy supported the hypothesis of a chemical effect by providing further insights into the osteogenic activity of dOBs seeded onto different polysaccharide scaffolds. Raman spectroscopy of the as-synthesized scaffolds revealed differences in saccharide components, including higher contents of sucrose and dextran in the FD-NanoClip fiber-type scaffold. ATR-FTIR spectroscopy then showed that the bone quality parameters mineral-to-matrix ratio was appreciably lower for dOBs seeded onto the sheet-type scaffold as compared to the fiber-type one. One possible explanation for the trend of the former quality parameter might be the difference in concentration of easily assimilable saccharide structures that provides a quick source of energy to osteoblasts. Glucose rings are used by osteoblasts for energy production through oxidative phosphorylation in the mitochondrial matrix. This process generates reduced coenzymes that lead to a transfer of free electrons in the inner mitochondrial membrane and finally to molecular oxygen, the terminal electron acceptor (Lee et al., 2017). An enhanced presence of disaccharides (e.g., sucrose) in the scaffold is thus expected to accelerate the energy metabolism in osteoblasts by more promptly providing isolate glucose rings as compared to long polysaccharide chains.

Regarding the recorded carbonate-to-phosphate ratios, all investigated scaffolds showed three- to fourfold higher values (i.e., 0.7–1.0) as compared to average values reported for both cancellous and cortical bone in healthy subjects (Figueredo et al., 2012). In a previous study (Horiguchi et al., 2019), the *in vitro* osteogenic performance of KUSA-A1 mouse mesenchymal cells seeded onto the same FD-NanoClip fiber-type was tested and atelocollagen sheet-type scaffolds are investigated in the present study. In both cases, the bone marrow stromal stem cell line differentiated into osteoblasts and abundantly produced bone tissue with an almost negligible content of calcium carbonate. A comparison with the present data thus suggests that the high value of carbonate-to-phosphate ratios, as recorded in this study, rather represents an intrinsic behavior of the dOBs directly converted from HDFs than being directly attributable to the chemistry of the used scaffolds. A high percentage of carbonate in the calcified bone matrix is related to high solubility by acid. It may reduce the strength of the transplanted calcified matrix, but it makes the transplanted scaffold easily absorbed by osteoclasts. This indicates easiness in being replaced by new intrinsic bone. Artificial scaffolds that have been used in a clinical trial for bone regeneration have normally lower carbonate-to-phosphate ratios. So, they have enough strength, but this characteristic involves the disadvantage that they are too hard to be resorbed by osteoclasts and to be replaced into bone tissue. As a result, they remain for a long time in the transplanted part without being replaced. This study shows that one could modulate and adjust the nature of transplanted scaffolds depending on the seeded cells, which is another advantage of FD-NanoClip gel.

## Conclusion

This study reported the osteogenic performance of two FD-NanoClip polysaccharide scaffolds with the same pore structure but different morphologies (sheet vs. fiber) in comparison with a sheet-type atelocollagen scaffold with a similar pore structure. All scaffolds were tested against the same dOBs, a cell line directly converted from HDFs, and proved capable of biologically active effects. Both types of FD-NanoClip scaffolds showed a higher degree of calcification at 21 days in culture as compared to the atelocollagen scaffold. However, the one with the fiber-type morphology was more than twice as effective as the sheet-type one. This difference was mainly attributed to a 5.5 higher surface area per unit volume of the fiber-type as compared to the sheet-type morphology. Vibrational spectroscopy assessments revealed subtle differences in the polysaccharide structures of the scaffolds, which nevertheless led to fundamental differences in bone quality. The FD-NanoClip fiber-type scaffold enabled the formation of bone tissue with a higher mineral-to-matrix ratio as compared to the sheet-type one. Although this result was in agreement with the assessments of calcification behavior in showing an enhanced formation of bone mineral, it could be linked to the Raman spectroscopic finding of a higher concentration of easily assimilable disaccharides as a quick source of energy rather than to morphological differences. A high carbonate-to-phosphate ratio was a common characteristic of all studied scaffolds, independent of their polysaccharide and atelocollagen structure. Since this characteristic was not found in previous studies of the same scaffolds tested vs. osteoblasts differentiated from a different mesenchymal cell line, it was explained as an intrinsic property of the HDF-dOB cell line rather than being directly related to scaffold chemistry.

In summary, this study clearly shows the possibility to eliminate the risk of tumorigenesis in the preparation of HDF-dOB cell line to be seeded onto high-performance, fully biodegradable FD-NanoClip scaffolds free of xenogenic proteins. However, the present data also stress the importance of monitoring bone quality parameters in order to finely adjust osteoblast physiology to produce tissue with structural characteristics comparable with those of human bone.

## Data Availability

The raw data supporting the conclusion of this article will be made available by the authors, without undue reservation.
